# A Concept for Smartphone-Based Emergency Flight Data Indication Systems in Light Aircraft

**DOI:** 10.3390/s26113368

**Published:** 2026-05-26

**Authors:** Jan Kaczyński, Paweł Rzucidło

**Affiliations:** Faculty of Mechanical Engineering and Aeronautics, Rzeszow University of Technology, Al. Powstańców Warszawy 8, 35-029 Rzeszów, Poland; 170103@stud.prz.edu.pl

**Keywords:** smartphone, light aircraft, emergency indications, flight data, MEMS

## Abstract

This paper explores the feasibility of using smartphones as emergency flight data indication systems in light aircraft. The presented solution may be applied in potential situations such as failures of the vacuum system or the gyroscopes driving analog instruments, as well as electrical power failures in aircraft equipped with digital avionics. Such failures may lead to the loss of essential flight information, significantly increasing pilot workload and conceivably compromising flight safety. The analysis was based on simulations conducted in a computational environment utilizing a custom-developed model. An experimental measurement flight using the MP-02A “Czajka” aircraft was conducted to collect real flight data for integration into a computational model. During the test flight, the aircraft was deliberately maneuvered into various attitudes and flight conditions to evaluate the model’s performance across the widest possible range of operating states. A smartphone mounted in the cockpit recorded sensor data, including accelerometer, gyroscope, magnetometer, and GPS information. The results demonstrated that key flight parameters can be accurately determined using only data recorded by a smartphone. For example, the determined pitch angle values during stall maneuvers deviate from the reference values by no more than 5°. The proposed solution shows significant potential for further development and practical implementation as a supplementary system to assist pilots during in-flight emergencies.

## 1. Introduction

Technological development over the past few decades has completely transformed the modern world. Undoubtedly, one of the most significant advances can be observed in the field of mobile telephony and mobile-based communication. The acquisition of smartphones equipped with a wide range of sensors and measurement devices is now feasible for almost everyone, including general aviation pilots.

The concept of using mobile devices in aviation navigation, such as tablets or smartphones, emerged relatively recently. At the turn of the first and second decade of the 21st century, projects began to develop that focused on creating applications or programs that would serve as comprehensive and fully sufficient tools for flight planning. Applications like, for example, *SkyDemon* [1], gathered in place data about current and planned airspace usage, issued NOTAMs (Notice to Air Missions), provided data on weather conditions, and included publications and aeronautical maps. A natural next step was the integration of these applications with satellite navigation systems, such as GPS. The authors of a study published in 2013 [2] demonstrated the potential of using smartphones in aviation navigation back then. Currently, the most popular applications in this field are *ForeFlight* [3] and, as mentioned earlier, *SkyDemon*. The International Civil Aviation Organization (ICAO), in document A41-WP/501, points to a growing trend of using electronic portable devices during flights [4]. A study conducted to assess the frequency of cockpit use of portable mobile devices not approved by company policy among commercial pilots [5] revealed that eighteen out of twenty respondents declared frequent or occasional use of such devices. It can be therefore assumed that among general aviation pilots—where internal regulations governing the use of mobile devices on board are usually absent—the percentage of such users is even higher. This is evidenced by the fact that many commercial smartphone apps are being developed specifically for use during flight [1,3,6,7,8].

Nowadays, one of the most common navigation methods among general aviation pilots is the use of devices supporting Global Navigation Satellite Systems (GNSSs). This method proves particularly useful during flights over vast areas lacking distinctive navigation landmarks, in the vicinity of densely distributed controlled airspaces, or under adverse weather conditions. However, the potential inherent in portable mobile devices allows consideration of their application in emergency situations, such as failures of onboard instruments responsible for providing spatial orientation and heading information. For example, the authors of paper [9] developed the Stratux system, consisting of a Raspberry Pi microcontroller, a GPS module, and an IMU (Inertial Measurement Unit) equipped with low-cost MEMS (Micro-Electro-Mechanical Systems) sensors [10,11,12]. In this setup, a smartphone was used to record flight parameters, which were subsequently compared with readings from certified Garmin G1000 EFIS avionics [13]. The study [9] focused on the Stratux system’s capability to detect aircraft bank angles exceeding 45° and on the development and testing of improved post-processing algorithms.

Another example of using mobile applications for monitoring and recording flight parameters is the GDL system [13]. This solution provides ready-to-use flight parameter information to a mobile application. In this configuration, the smartphone does not act as a measuring device; it only uses data transmitted from the aircraft’s avionics via Bluetooth.

This paper presents the course of an experiment aimed at demonstrating the feasibility of using smartphones as autonomous emergency indication systems. Test measurements were performed using a smartphone whose internal MEMS sensors collected data related to angular velocities, linear accelerations and local magnetic field information [14]. A simple computational model was also proposed to transform the collected raw data into usable flight parameters, such as attitude angles, heading, altitude and ground speed. These parameters are critical during emergency and distress situations, and the availability of an alternative source of such information may contribute to improving the overall safety of air operations.

The most significant novel elements of this work include an original computational model based on complementary filters, which performs data fusion from MEMS sensors and GNSS receiver data provided by a smartphone. This model was designed with the limited computational power of the hardware platform (a common smartphone) in mind. The data for experimental verification were recorded during an actual flight of the MP-02A ‘Czajka’ aircraft. Processed data from an iPhone 13 (collected using the MATLAB 2025b Mobile app) were directly compared with data from a professional Dynon SkyView D-700 digital avionics system [15]. This allowed for a precise error assessment during real-world flight conditions, such as high-bank turns or stalls. The study provides unique data on the behavior of smartphone-based flight indicators during dynamic maneuvers, such as stalls in various configurations (with flaps extended and retracted).

## 2. Legal Aspects of Smartphone Use in General Aviation Aircraft

The use of mobile devices by pilots causes significant controversy. There are concerns about the occurrence of dangerous situations caused by pilot distraction due to the use of portable electronic equipment. In cases where technology in a given area is evolving at a rapid pace, the legal regulations governing and defining the rules for its use are created too slowly and are often outdated by the time they come into effect. The most used solution so far regarding mobile devices is the implementation of internal regulations within airline operators, setting the usage of these devices.

The European Union Aviation Safety Agency (EASA) defines Portable Electronic Devices (PED) as devices “typically but not limited to consumer electronics, brought on board the aircraft by crew members, passengers, or as a part of the cargo that are not included in the approved aircraft configuration” [16]. This is defined in the Guidance Material 1 (GM1) supplementing the regulation contained in Part-NCO.GEN.125. Moreover, under this definition, it also includes any device that uses electrical energy, which can be provided from internal sources, such as batteries, or drawn from specific electrical outlets in the aircraft. The EASA divides PEDs into two categories:

- Transmitters that emit radio waves unintentionally;
- Transmitters that emit radio waves at specific frequencies as their intended operation.

The first category includes devices such as audio and video players, cameras, and basic calculators. The second category regards mobile phones, including smartphones, portable radio transmitters, so-called walkie-talkies, or laptops. The Guidance Material for Part-NCO.GEN.125 also defines the “off” status of PEDs and emphasizes that, when turned off, many of them are not fully disconnected from power, as internal components like clocks continue to operate and can still be a source of interference. Additionally, GM2 to Part-NCO.GEN.125 indicates that PEDs can interfere with electronic components on board the aircraft, which may imply potential failures or malfunctions in certain systems. In point (c) of GM2, there is also a warning about an increased risk of such interference in light aircraft, due to the proximity of individual components and their limited shielding in such designs.

The Part-NCO.GEN.125 regulation states that “The pilot-in-command shall not permit any person to use a portable electronic device (PED) on board an aircraft, including an electronic flight bag (EFB), that could adversely affect the performance of the aircraft systems and equipment or the ability of the flight crew member to operate the aircraft” [16]. However, there are no legal regulations that explicitly and directly prohibit or allow pilots to use mobile devices during flight in light aircraft. The possibility of their use can be considered in the context of the airworthiness certificate issued for a particular aircraft. Point 3.2.1 of Chapter 3 of Annex 8 to the Convention on International Civil Aviation—known as the Chicago Convention—states that “A Certificate of Airworthiness shall be issued by a Contracting State on the basis of satisfactory evidence that the aircraft complies with the design aspects of the appropriate airworthiness requirements” [17]. It must therefore be stated that the legally permissible use of mobile-type devices, such as smartphones or tablets, is contingent upon the specification of that device being included in the application for the issuance of the airworthiness certificate. Consequently, it is reasonable to assert that the civil aviation authority of the respective country, as the superior body issuing the airworthiness approval for a given aircraft, decides whether the given devices meet the requirements that qualify them for use during flight.

Laws evolve and are continually subject to amendments and updates. It cannot be ruled out that in the future, a regulation will be adopted that prohibits the use of mobile devices during flight. Nonetheless, if, during a flight, an emergency situation arises, and the use of a smartphone as a remedial measure contributes to the safe conclusion of the flight operation, the commander is not criminally liable for its use. This is carried out in the name of the greater good, which is saving the lives of all on board. Thus, there is a justification, i.e., a circumstance that excludes the unlawfulness of the act—in this case, the state of necessity. In Polish law, this is defined in paragraph 1 of Article 26 of the Penal Code and translated as “No offence is committed by someone who acts to avert an imminent danger threatening and legally protected good, if the danger cannot be avoided in any other way, and the good sacrificed is of lesser value than the good being preserved” [18].

The legal analysis must also address the issue of mounting smartphones in the cockpit. The common practice in general aviation is the use of dashboard mounts that can be quickly removed, where the mobile device is placed. They are mainly used as navigation aids, but they do not constitute the primary or essential equipment for conducting the flight. Any other method of mounting can be considered a modification—change in the aircraft’s structure. Modifications in aircraft design in aviation can be divided into two categories—minor and major changes. This is defined by the provision in point 21.A.91 Part D of Annex 1 to Commission Regulation (EU) No. 748/2012 [19]. According to it, “A ‘minor change’ is one that has no appreciable effect on the mass, balance, structural strength, reliability, operational characteristics, noise, fuel venting, exhaust emission, or other characteristics affecting the airworthiness of the product”. Changes that go beyond the definition of a minor change are considered major. Placing PEDs in the aforementioned dashboard mount, which does not interfere with the aircraft’s structure, systems, or flight characteristics, does not meet any of the definitions given above. Therefore, there are no legal grounds prohibiting such practice.

## 3. Research Environment and Plan of Experiment

**Figure 1:**
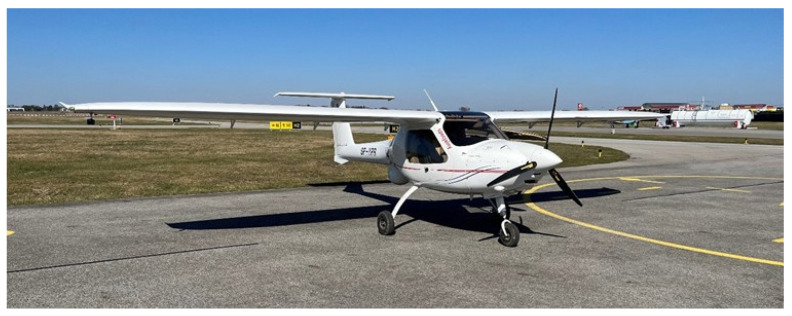
The MP-02A “Czajka” aircraft, registration SP-YPR.

The experiment aimed at investigating the feasibility of using a smartphone as an emergency flight data indication system was divided into two stages. The first stage consisted of recording real flight parameters during a test flight carried out using an MP-02A “Czajka” aircraft. “Czajka” is a light, special category aircraft manufactured by the Aero-Kros company (Krosno, Poland) and it is shown in Figure 1. It features a high-wing configuration and is powered by a Rotax 912ULS engine with an output of 93.5 kW.

The EFIS Dynon SkyView D-700 system (manufactured by Dynon Avionics company; Woodinville, Washington, DC, USA) installed in the aircraft serves as a source of information for the pilot, providing data on roll, pitch, altitude, aircraft speed, and current engine parameters. The Dynon D-700 system includes a function that allows recording flight state data onto removable memory storage devices. After completing the measurement flight, this function was used and the data were transferred to a portable USB drive. These data served as a reference source against which the results of later simulations were compared. The accuracy of these data, due to compliance with certification requirements, is ±1° for roll and pitch angles under static conditions and ±2.5° under dynamic conditions. The allowable heading errors are ±2° and ±4°, respectively [20].

The measurement flight was conducted from the Rzeszów Airfield (ICAO code: EPRJ). This airfield is located in close proximity to the international airport Rzeszów–Jasionka (ICAO code: EPRZ) and it is equipped with two runways—one paved and one grass—aligned along magnetic headings of 083–263°. The airfield is home to the Aviation Training Centre operating at Rzeszów University of Technology. Its proximity to a major commercial airport requires a flight plan to be filed for each intended air operation.

The measurement flight involved a series of maneuvers performed in a flight training area outside the airport’s controlled zone (CTR). These included turns with specified bank angles during climb, descent, and level flight. Data were also recorded during stall maneuvers.

During the flight, data were collected using an Apple iPhone 13 smartphone. For the purpose of the study and to facilitate the calibration process, the smartphone was mounted in a fixed position. Data recorded by the phone’s internal sensors—including angular velocities, linear accelerations, and magnetic field intensity—were logged for each timestamp in matrix form using a MATLAB mobile application. This application allows the user to select which internal sensors of the phone are to be included in the data recording process.

**Figure 2:**
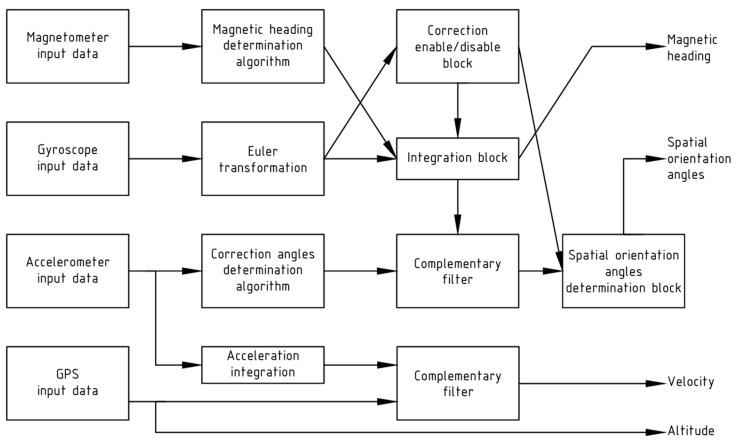
Block diagram of the simulation model.

Due to the use of the aforementioned application, the second part of the experiment consisted of performing appropriate simulations in MATLAB Simulink. This environment is user-friendly and enables the simple creation of desired simulation models. By using extensions such as the Aerospace Toolbox, visual representation of the obtained simulation results is also possible. The developed simulation model was divided into eight subsystems, each responsible for a specific function. Among them were, for example, subsystems for calculating roll angles and determining magnetic heading. All subsystems were integrated through the use of global variables within the simulation model. Figure 2 presents the block diagram illustrating the data flow in the developed model.

**Figure 3:**
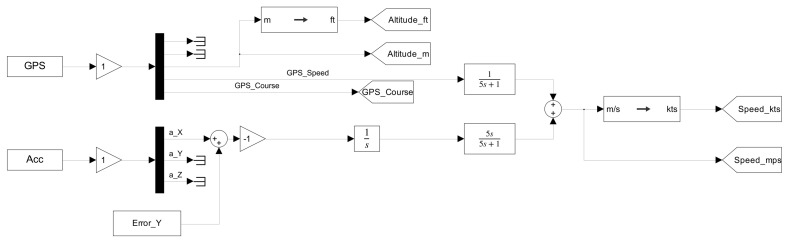
Block diagram of Velocity and Altitude Subsystem.

The Velocity and Altitude Subsystem acquires data from two sources: namely, measurements from the smartphone’s MEMS accelerometer and information from the GPS, which provides ground speed and altitude. The structure of this subsystem is illustrated in Figure 3.

Due to the inability to log barometer data from the smartphone within the MATLAB application, the process of determining the altitude profile does not have a more elaborate form. The velocity is determined by applying a complementary filter [21,22] that fuses data from the GPS and the MEMS accelerometer. The useful acceleration values are recorded along the aircraft’s X-axis, and after being read, the zero-offset error is compensated. The signal, once corrected for this bias, is integrated to yield successive velocity values. This process is depicted in Equation (1). As a result of signals being processed by the complementary filter, a velocity time series is obtained with a higher sampling frequency than the GPS system alone allows.

(1) $$v\left(t\right)=\int_{0}^{t}a\left(t\right)\mathrm{dt}$$

v(t)—velocity value at time t;

t—time;

a(t)—acceleration at time t.

**Figure 4:**
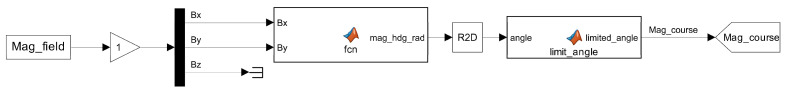
Block diagram of Magnetic Course Subsystem.

The process of determining the magnetic course also takes place within a dedicated subsystem and requires reading data only from the smartphone’s magnetometer. The structure of this subsystem is shown in Figure 4.

The read signal, containing the necessary data, is passed to a *MATLAB Function* block, which allows for the implementation of a custom algorithm that is represented by Equation (2):

(2) $$\mathrm{Ma}g_{\mathrm{course}}\;=\;\mathrm{atan}2\left(\frac{{-B}_{Y}}{B_{X}}\right)$$

$B_{X}$—magnetic induction along the X-axis;

$B_{Y}$—magnetic induction along the Y-axis.

The computed magnetic course is initially in the range of [−180°, 180°]; however, the desired range is [0°, 360°]. This is achieved by using the algorithm provided below. The signal, once confined within desired bounds, is stored as a global variable and used in the Gyro Angles and Final Course Subsystem.
function limited_angle = limit_angle(angle)     % Function that limits the angle range to 0–360 degrees     % Increasing the angle if it is less than 0     while angle < 0       angle = angle + 360;     end     % Decreasing the angle if it is greater than or equal to 360     while angle >= 360       angle = angle − 360;     end     limited_angle = angle; end

**Figure 5:**
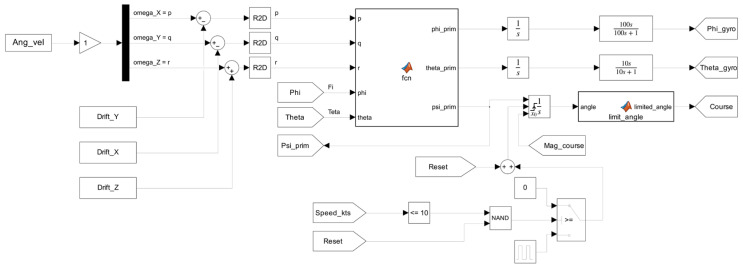
Block diagram of Gyro Angles and Final Course Subsystem.

The structure of the Gyro Angles and Final Course Subsystem is shown in Figure 5.

Similarly to velocity determination, a correction for the zero-offset bias is applied to the gyroscope input data, which, after integration, induces a drift error. Subsequently, the units are converted from radians per second to degrees per second. Within the *MATLAB Function* block, in addition to the angular velocities, the calculated final pitch and roll angles are also fed back. The algorithm contained in the block determines the derivatives of the Euler angles, which correspond to the readings of the aircraft’s AHRS system. These derivatives are defined by the following equations:

(3) $$\dot{\Phi }\;=\;P\;+\;Q\sin\Phi \tan\theta \;+\;R\cos\Phi \tan\theta$$

(4) $$\dot{\theta }=Q\;\cos\phi -\;R\sin\phi$$

(5) $$\dot{\psi }=Q\frac{\sin\phi }{\cos\theta }+R\frac{\cos\phi }{\cos\theta }\;$$

P,Q,R—angular velocity of roll, pitch, and yaw in the aircraft-fixed frame;

ϕ, θ, ψ—Euler angles.

The computed derivatives of the pitch and roll angles are integrated, and the signals then pass through a high-pass filter, thus producing the gyroscopic angles. The integration of the derivative $\dot{\psi }$ is carried out with a reset mechanism, which, upon meeting certain conditions, sets the initial value according to the currently determined magnetic course. The output from the integrator is verified using the previously shown angle-limiting algorithm. In this way, the final course is obtained.

**Figure 6:**
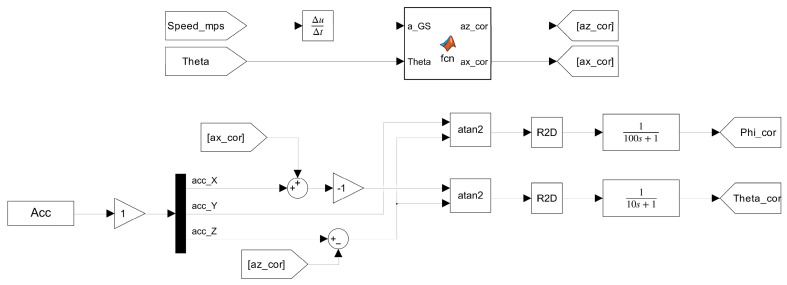
Block diagram of Correction Angles Subsystem.

The correction angles provide a good approximation of the aircraft’s spatial orientation angles, provided that the flight is in a steady state. The structure of the subsystem in which these angles are determined is shown in Figure 6.

The inputs to this subsystem are the accelerations measured by the smartphone’s MEMS accelerometer. For signals recorded along the X- and Z-axes, an adjustment is applied to account for errors resulting from changes in the aircraft’s velocity. Based on Equations (6) and (7), the correction angle values are computed:

(6) $$\phi_{\mathrm{cor}}\;=\;\mathrm{atan}2\left(\frac{a_{Y}}{a_{Z}}\right)$$

(7) $$\theta_{\mathrm{cor}}=\mathrm{atan}2\left(\frac{{-a}_{X}}{a_{Z}}\right)$$

$a_{X},\;a_{Y},a_{Z}$—acceleration measured along the aircraft’s X, Y, Z-axes.

The unit of the calculated angles is then converted from radians to degrees, so that it is consistent with the other signals in the simulation model. Before leaving the subsystem, the data stream passes through low-pass filters to reduce high-frequency components. In this way, the output signal undergoes the first stage of complementary filtering.

The gyroscopic and correction spatial orientation angles are fused using complementary filters in the remaining subsystems of the simulation model [21]. The time constants of the complementary filters were tuned by minimizing the Mean Absolute Error (MAE), as in [22]. This approach yields the final values of these parameters.

**Figure 7:**
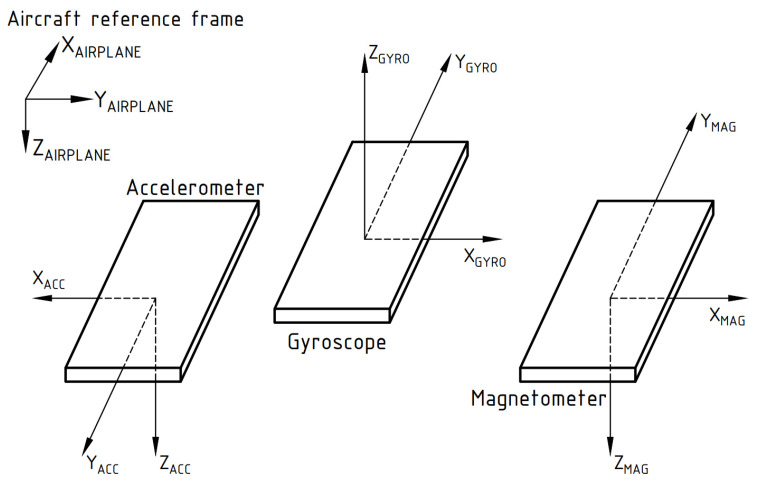
Spatial arrangement of the main measurement axes of the smartphone’s MEMS sensors.

Due to the file format in which the MATLAB application outputs measurement results, it was necessary to create a dedicated script to implement the collected data into the simulation model. At this stage, it was also necessary to include correction algorithms in the script. This requirement arose from the spatial arrangement of the sensors within the smartphone. Each sensor was positioned in such a way that measurements along a given aircraft axis were recorded along different axes in each sensor. The correction algorithm therefore unified all sensor axes and transformed them into the aircraft reference axes. The spatial orientation of the sensor axes relative to the aircraft’s principal axes is presented in Figure 7.

## 4. Results

The starting point for considering the possibility of using smartphones as an emergency flight data indication system in light aircraft is the results of the performed simulations. The output data from the simulation model consist of time histories of selected flight parameters, such as roll angle, pitch angle, and magnetic heading. These were presented graphically together with the corresponding reference data recorded by the Dynon SkyView D-700 EFIS system. This allows for a visual comparison of the characteristics of the signals and an assessment of the accuracy of the data produced by the simulation model.

### 4.1. Roll and Pitch Angle Analysis

**Figure 8:**
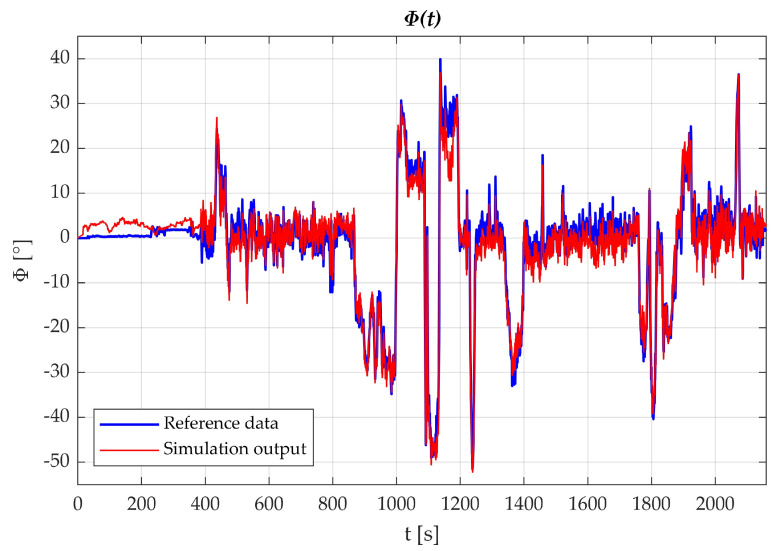
Time histories of the roll angle.

The time histories of the roll angle are presented in Figure 8. The data determined by the simulation model are marked in red. The reference data from the Dynon SkyView D-700 system, on the other hand, are marked in blue.

It can be observed that the nature of both signals is very similar, and the values calculated by the simulation model do not noticeably deviate from the reference data. It is easy to identify the individual phases of the flight, such as turns with a specific bank angle or level flight. Despite the clear similarity between the reference signal and the one from the simulation, certain discrepancies can be observed at specific moments. Particular attention is drawn to the zero-roll angle calculated in the initial segment of the graph. The cause of this may be an uncompensated zero-offset error of the MEMS accelerometers in the model’s angle correction subsystem, or due to uneven lateral balancing of the aircraft.

**Figure 9:**
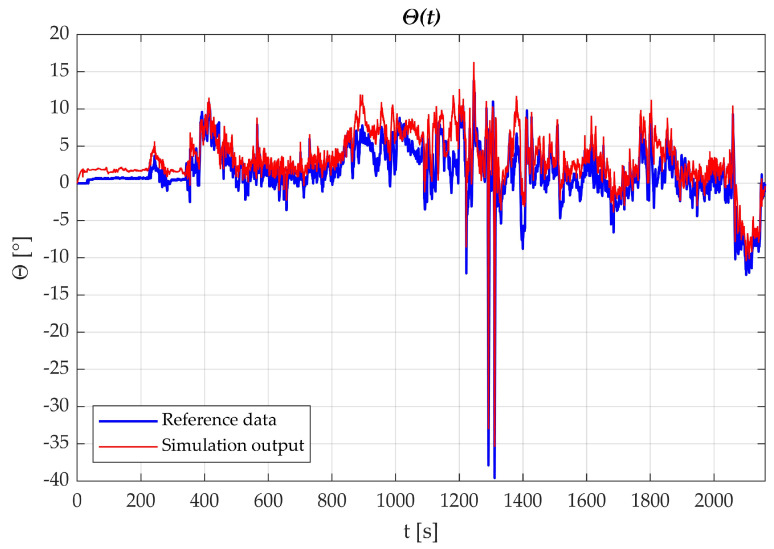
Time histories of the pitch angle.

The time history of the pitch angle is presented in Figure 9. The same color convention regarding the source of the signals has been maintained.

The pattern of changes in the signal determined by the simulation accurately reflects the course of the reference data, but it is clearly offset relative to them. The cause in this case is the lack of zero-offset correction for the MEMS accelerometers in the model’s angle correction subsystem. Non-zero acceleration values recorded along the aircraft’s X-axis led the model to return positive pitch angle values, causing the aforementioned overestimation.

**Figure 10:**
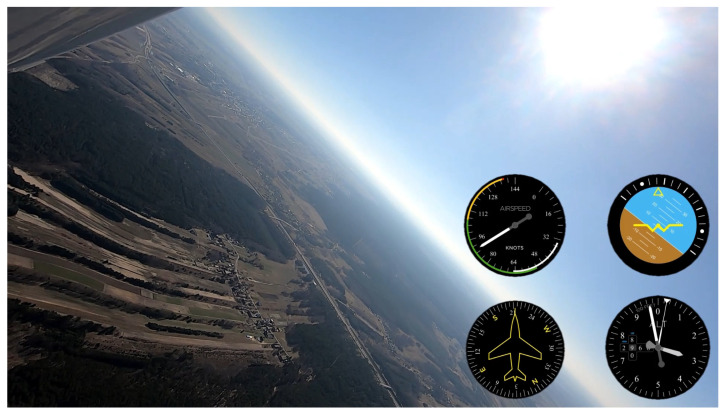
Measurement flight frame with overlaid flight instruments during a 30° bank left turn in level flight.

Figure 10 shows a frame from a video recorded during a left turn with a 30° bank angle at a constant altitude, with onboard instruments overlaid. These instruments display the flight parameter values at that moment, as determined by the simulation model. At the moment shown, the roll angle slightly exceeds the intended bank angle. It is difficult to determine whether this is caused by the inaccuracy of the simulation model or by pilot inaccuracy. The positive pitch angle shown in the figure, however, does not indicate a climb; rather, it is necessary to provide the appropriate lift for level flight.

By analyzing the graphs presented in Figure 8 and Figure 9, the maneuvers that defined the specific phases of the flight were identified. Knowledge of the time signatures marking the start and end of each phase determines its total duration. The number of samples with useful data recorded during its time is given by Equation (8):

(8) $$n\;=\;t_{\mathrm{phase}}\;\cdot \;\nu_{\mathrm{sensor}}$$

n—number of samples in a given flight phase;

$t_{\mathrm{phase}}$—total duration of the given phase;

$\nu_{\mathrm{sensor}}$—sensor sampling frequency.

The adopted number of samples n resulted from the duration of the analyzed flight segments and a sampling frequency of 100 Hz. It ranged from 4100 samples for the initial climb phase to 36,000 samples for the transit to the flight training area. Knowledge of the number of samples in each phase permitted the determination of the average value of the given parameter, in accordance with Equation (9):

(9) $$\bar{x}\;=\;\frac{1}{n}\sum_{i=1}^{n}x_{i}$$

$x_{i}$—the value of an individual sample.

The parameter that allows for the assessment of the dispersion of a given value around the arithmetic mean is the standard deviation. Its value was calculated for each phase according to Equation (10):

(10) $$\sigma \;=\;\sqrt{\frac{1}{n\;-\;1}\sum_{i=1}^{n}{\left(x_{i}\;-\;\bar{x}\right)}^{2}}$$

For each phase, the maximum difference between the parameter value determined by the simulation model and the reference parameter was also identified. To this end, a vector was created whose successive elements specified the aforementioned difference for each sample. This procedure is defined using Equation (11):

(11) $$\Delta x\;=\;\left|x_{i}\;-\;x_{\mathrm{ref}}\right|$$

$x_{\mathrm{ref}}$—reference value corresponding to the sample $x_{i}$.

The maximum value in such a defined set allows for the determination of the maximum error in a given flight phase. This process is described by Equation (12):

(12) $$\Delta x_{\max}\;=\;\max\left(\Delta x\right)$$

In order to determine the average distance between the values predicted by the model and the actual values, the root mean square error (RMSE) was calculated, as defined using Formula (13).

(13) $$\mathrm{RMSE}=\sqrt{\frac{1}{n}\sum_{i=1}^{n}{\left(x_{i}-x_{\mathrm{ref}}\right)}^{2}}$$

$x_{\mathrm{ref}}$—the reference value for an individual sample.

In Table 1, statistical data for the roll angle are summarized. The mean value of the data determined by the simulation model differs from the mean of the reference parameters by no more than 2° in most cases. An exception is the right turn in level flight with a roll angle of 30°, where the difference amounted to as much as 5.78°. This phase also recorded the largest instantaneous difference between the measurement signals and the reference, as well as the RMSE. The standard deviation values reach similar levels for each phase, both for the measurement and reference data. This points to the statistically similar characteristics of both the reference and the smartphone-based calculated signals.

The statistical data for the pitch angle are compiled in Table 2. The largest difference in mean value was recorded for the left turn in level flight with a roll angle of 30°, amounting to 4,1°. The largest instantaneous difference was also observed during a turn in level flight, this time to the right, with a roll angle of 30°. The largest error values, for both roll and pitch angles, occur at high bank angles. This is the primary limitation of the presented solution. In an emergency, the pilot should be aware that during abrupt maneuvers, the attitude error may be significant, which is unfortunately a typical characteristic of solutions utilizing low-cost MEMS sensors. The standard deviation values, both for the calculated roll angles and the reference ones, are very similar, which once again indicates proper dynamics in the recording of the occurring changes.

In a system based on complementary filters, without full dynamic compensation (which is very difficult to achieve on a flying object), an increase in error as the maneuver deepens is expected (please note the errors during turns with a 30-degree bank in Table 1 and Table 2). Even with such an error, the device still provides the pilot with critical information regarding the attitude as well as roll and pitch trends in the event of a total loss of onboard instruments and the lack of a visible horizon line.

### 4.2. Magnetic Heading Analysis

**Figure 11:**
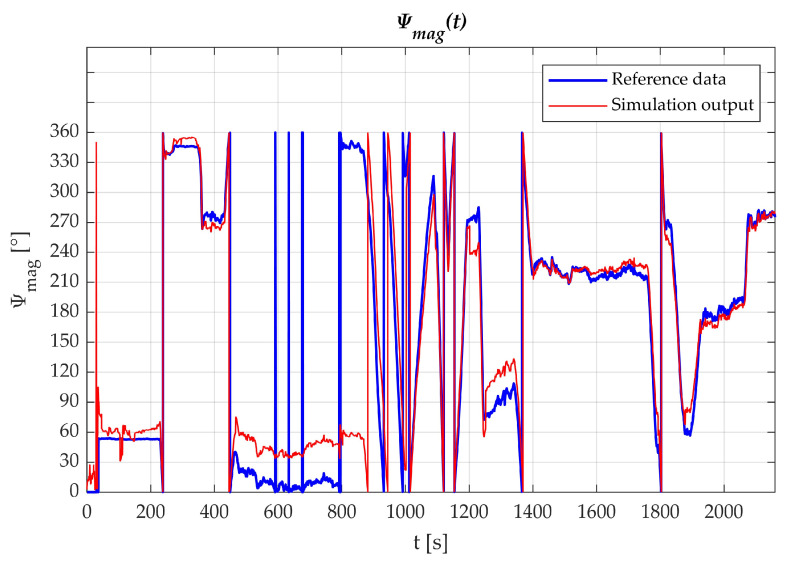
Time histories of the magnetic heading.

To unambiguously determine the three-dimensional position of the aircraft, in addition to the roll and pitch angles, it is also necessary to define the direction in which the aircraft is moving. In this case, it is the magnetic heading. The comparison of the heading signals obtained from the model and the reference is shown in the chart in Figure 11.

Its elements that immediately catch the eye are the abrupt jumps, represented by vertical segments connecting values from 0° to 360°. Their presence is not a sign of an error, but rather a consequence of the chosen method of representing this parameter. Magnetic heading is a cyclic quantity, and its values range within the interval from 0° to 360°. Reaching one boundary of this range causes a transition to the other end, depending on the direction of the change. On the plot, this appears as an abrupt jump, although in reality, it is a continuous and correct change in the parameter’s value.

The time history of the magnetic heading exhibits a very similar pattern of changes compared to the reference signal. The highest accuracy can be observed during flight phases where a relatively constant heading is maintained, which are represented by the horizontal segments of the signal on the graph. The greatest deviation of the calculated values from the reference values was noted between 470 s and 850 s, the timestamp of the measurement. Such significant discrepancies, reaching around 60° in some cases, occur only during flight with a heading approximately north of northeast. The magnetic deviation caused by moving control system components (pushrods) located in the tunnel, over which the smartphone was positioned during flight, could also have had a significant impact on magnetic heading estimation errors. The method used in the computational model to determine the magnetic heading proves effective, especially during small or slow changes. During rapid transitions, it is clearly visible that the computed heading does not reach zero degrees and undergoes a sudden jump. Although this type of error does not pose a major risk of misinterpreting the data by the pilot, it is advisable that turns and the associated heading changes be performed as smoothly and gradually as possible.

**Figure 12:**
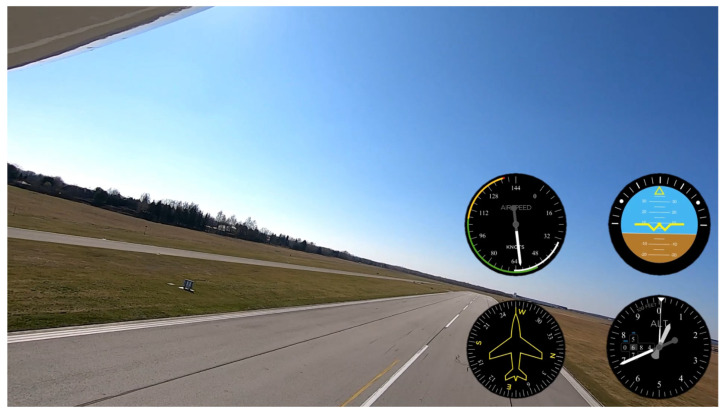
Measurement flight frame with overlaid flight instruments during initial climb (the attitude indication appears misleading because the camera was mounted perpendicular to the wing with a positive dihedral, which tilts the recorded image plane relative to the true horizon plane and makes the visible horizon differ from the attitude indicator).

Similarly to what was carried out with the pitch and roll angles, Figure 12 presents a frame from the video recorded during the measurement flight, in the phase of initial climb. One of the instruments visible in the photo is a compass, which displays the magnetic heading determined by the computational model. At the visible moment, right after takeoff, the aircraft’s actual heading aligns with the direction of the runway axis, in this case 263°. Thus, the calculated magnetic heading visible on the compass is approximately correct, and the error does not exceed 5°.

### 4.3. Ground Speed and Altitude Analysis

For the safety of the flight, the most critical parameters are speed and altitude. Speed, depending on how it is determined, can be related to the current value of the lift force that allows the aircraft to stay in the air. Constant monitoring of the aircraft’s altitude is essential to avoid violating the boundaries of controlled airspaces or colliding with the ground, which is particularly important in mountainous terrain.

**Figure 13:**
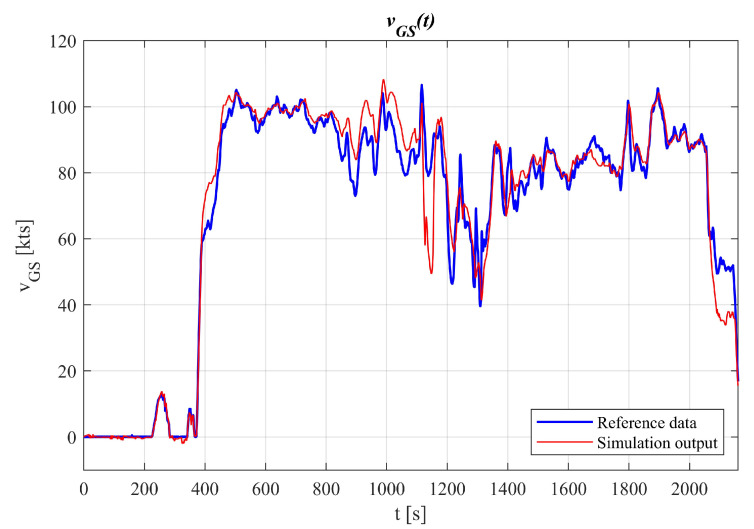
Time histories of the ground speed.

Figure 13 shows the aircraft’s ground speed as a function of time. It was determined by fusing data from the GPS and the MEMS accelerometer. This achieved a higher sampling rate than using the GPS alone. This is undoubtedly advantageous in situations of temporary loss of satellite reception, as the continuity of the signal is maintained. The resulting signal closely matches the reference one, preserving the character and dynamics of the changes. Distinct phases of the measurement flight are visible, such as taxiing to the runway threshold, stalls, and the final approach to landing. The determined RMSE of the ground speed, depending on the flight phase, ranged from 2.1 kt (cruise to the maneuvering area) to 18.3 kt for a 30-degree bank right turn.

Determining airspeed, which is so crucial for ensuring flight safety, appears to be an extremely difficult task using only a smartphone. In future work, the authors plan to address this problem by utilizing real-time weather data.

**Figure 14:**
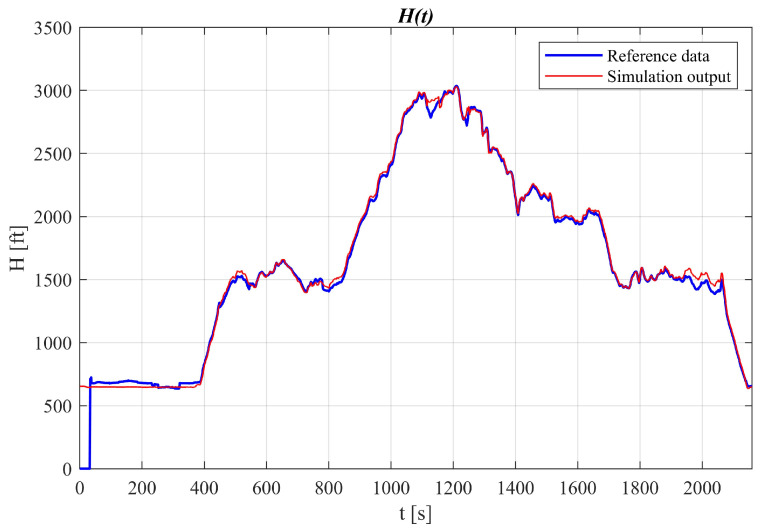
Time histories of the altitude.

The comparison of the altitude time series is shown in Figure 14. The signals determined using data from the smartphone rely solely on information obtained from the GPS system. This is due to the inability to record data in the mobile MATLAB application using the barometer integrated in the mobile device. It can be stated that there is a high consistency between the data returned by the computational model and the reference data. The determined RMSE of the altitude, depending on the flight phase, ranged from 20 ft (back to the airport cruise leg) to 68 ft for a 30-degree bank left turn.

Although the flight altitude data recorded by the smartphone are very close to the data obtained from the onboard avionics, it will be worth considering data fusion from accelerometers in future models. Such an approach will have a positive impact on the integrity of the obtained readings.

### 4.4. Stall Condition Analysis

**Figure 15:**
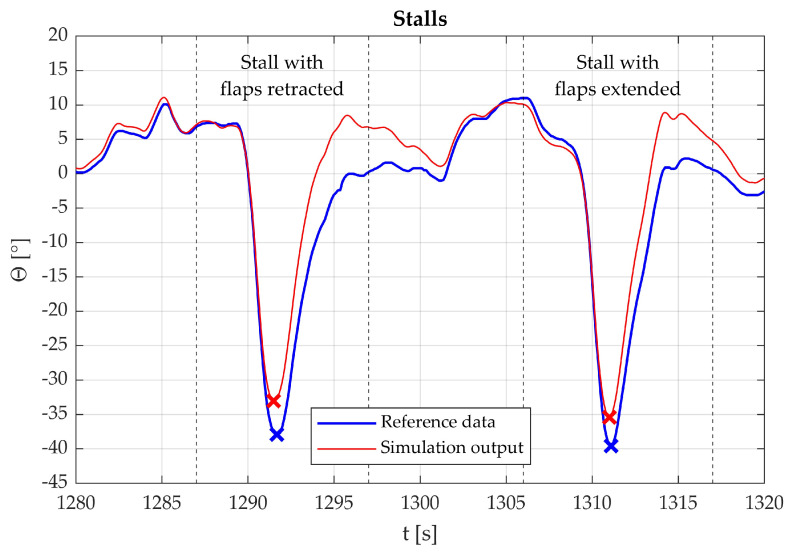
Time histories of performed stalls.

During stalls, as a result of exceeding the critical angle of attack, the generated lift decreases, and the total aerodynamic drag increases. The natural consequence of this is a dynamic pitching down of the aircraft’s nose toward the ground and thus a reduction in the pitch angle. Figure 15 presents a graph illustrating two stalls. One was performed in a clean configuration, with flaps retracted, while in the second, the flaps were extended to their maximum angle, which in the MP-02A “Czajka” aircraft is 48°.

In both cases, the data obtained using the computational model were overestimated compared to the reference data. The exact values at the moment of maximum pitch angle (symbol ‘x’ in Figure 15) are compiled in Table 3.

**Figure 16:**
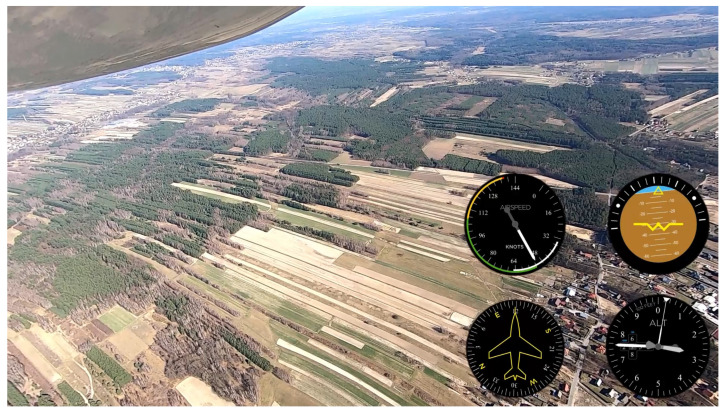
Measurement flight frame with overlaid flight instruments during a clean configuration stall.

The stall performed with the flaps fully extended resulted in a greater relative value of the pitch angle. Figure 16 presents a frame from the video recorded during the measurement flight, showing the moment of the stall with flaps retracted.

The artificial horizon visible in the figure indicates a pitch angle of 30°. This is caused by a limitation applied within it. This block, in the Simulink environment, is programmed so that exceeding the allowable values of the roll or pitch angle causes the indications to freeze at the maximum allowable values, which is what happens in this case.

## 5. Conclusions

The purpose of the experiment was to determine the feasibility of using smartphones as emergency flight parameter indication systems in light aircraft. The conducted analysis showed that the accuracy of the data recorded by the mobile device demonstrate the potential for emergency support as a supplementary system, provided the pilot is aware of the current technical limitations. This conclusion pertained to the specific device model used. However, it should be noted that significant discrepancies may exist between different smartphone models.

The paper included the methodology and a description of the test flight, during which the key measurements were carried out. They relied on the use of satellite navigation systems and micro-electro-mechanical system sensors, which were used to collect the data necessary for the analysis. The structure of the proposed computational model was developed based on complementary filters, which allowed for the assessment of their effectiveness. This was also a modification of the approach presented in already existing articles, where different data filtering methods from MEMS sensors were used.

Based on the conducted analysis, it was concluded that the proposed computational model structure and the solutions adopted within it enable achieving sufficiently high accuracy of the determined flight parameters. In most instances, the deviations and differences between the time series obtained from the simulation and the reference data are small enough to support the rationale for continuing the concept of developing the use of smartphones as emergency indication systems.

In the future, it is worth considering the implementation of more precise computational algorithms that account for, for example, the inclination of the Earth’s magnetic field, and to explore the impact of using more advanced data fusion methods, such as the Kalman filter. The research results presented in this paper refer to a single, selected model of a mobile device. In future work, we intend to conduct comparative studies on several representative smartphone models from various manufacturers. The promising results obtained from the simulations confirm the significance of developing a mobile application for general aviation pilots, which could additionally serve as a source of information in case the flight parameters cannot be read from onboard instruments.

## Figures and Tables

**Table 1:** Statistical analysis of the roll angle.

Flight Phase	Mean Value		Standard Deviation		Maximum Error	RMSE
	Recorded Data	Reference Data	Recorded Data	Reference Data		
Initial climb	2.80°	−0.20°	2.4426	2.4018	4.86°	3.09°
Cruise to the maneuvering area	0.83°	0.70°	2.9922	2.9656	5.14°	1.75°
Left turn during climb with a 15° bank angle	−20.61°	−20.76°	5.7634	5.0029	2.21°	1.01°
Right turn during climb with a 15° bank angle	16.20°	18.16°	5.2317	4.5287	4.02°	2.28°
Left turn in level flight with a 30° bank angle	−45.11°	−43.02°	3.3154	4.4249	7.37°	3.35°
Right turn in level flight with a 30° bank angle	22.06°	27.84°	4.8820	2.8435	12.05°	6.56°
Left turn during descent	−21.61°	−22.75°	5.2649	6.3410	3.81°	1.87°
Back to the airport cruise leg	−1.30°	0.76°	3.1020	2.8934	5.56°	2.41°
Final approach	2.06°	1.44°	3.1618	3.2476	2.63°	1.22°

**Table 2:** Statistical analysis of the pitch angle.

Flight Phase	Mean Value		Standard Deviation		Maximum Error	RMSE
	Recorded Data	Reference Data	Recorded Data	Reference Data		
Initial climb	8.08°	7.72°	1.3296	1.4434	2.08°	0.90°
Cruise to the maneuvering area	2.69°	1.48°	1.2843	1.5308	2.98°	1.36°
Left turn during climb with a 15° bank angle	8.35°	5.32°	1.5095	1.1262	5.24°	3.23°
Right turn during climb with a 15° bank angle	7.07°	5.33°	0.8593	1.5130	4.13°	2.29°
Left turn in level flight with a 30° bank angle	6.05°	1.95°	2.6510	2.4554	8.49°	4.78°
Right turn in level flight with a 30° bank angle	7.29°	3.80°	2.2668	2.2645	8.81°	4.65°
Left turn during descent	5.32°	1.90°	3.4026	3.4104	6.74°	4.02°
Back to the airport cruise leg	2.33°	0.57°	2.2195	2.3824	4.41°	1.99°
Final approach	−7.22°	−8.75°	1.2158	1.5896	2.90°	1.62

**Table 3:** Statistical analysis of the pitch angle during stall events.

Stall	Model Output	Reference Data	Difference
Flaps retracted	−33.05°	−37.90°	4.85°
Flaps extended	−35.42°	−39.60°	4.18°

## Data Availability

The original contributions presented in this study are included in the article. Further inquiries can be directed to the corresponding author.

## Abbreviations

The following abbreviations are used in this manuscript:

CTR	Control Zone
EASA	European Union Aviation Safety Agency
EFIS	Electronic Flight Instrument System
EU	European Union
GM	Guidance Material
GNSS	Global Navigation Satellite System
GPS	Global Positioning System
ICAO	International Civil Aviation Organization
IMU	Inertial Measurement Unit
MAE	Mean Absolute Error
MEMS	Microelectromechanical system
NOTAM	Notice to Air Missions
PED	Portable Electronic Device
RMSE	Root Mean Square Error
USB	Universal Serial Bus
